# Knockdown of RFC4 inhibits cell proliferation of oral squamous cell carcinoma *in vitro* and *in vivo*

**DOI:** 10.1002/2211-5463.13929

**Published:** 2024-12-13

**Authors:** Pengyue You, Di Wang, Zheng Liu, Shuzhen Guan, Ning Xiao, Haotian Chen, Xin Zhang, Lichuan Wu, Guizhen Wang, Haitao Dong

**Affiliations:** ^1^ Department of Stomatology, Peking Union Medical College Hospital Chinese Academy of Medical Sciences and Peking Union Medical College Beijing China; ^2^ Department of Clinical Laboratory, Peking Union Medical College Hospital Chinese Academy of Medical Sciences and Peking Union Medical College Beijing China; ^3^ State Key Laboratory of Molecular Oncology, Department of Medical Oncology, National Cancer Center/National Clinical Research Center for Cancer/Cancer Hospital Chinese Academy of Medical Sciences and Peking Union Medical College Beijing China; ^4^ Medical College of Guangxi University Nanning China

**Keywords:** immunotherapy, oral squamous cell carcinoma, proliferation, RFC4, WGCNA

## Abstract

Oral squamous cell carcinoma (OSCC) is the one of the most common types of malignant tumor found in the head and neck area. Replication factor C subunit 4 (RFC4), an oncogene active in various human cancers, has been rarely studied in OSCC. In the present study, bioinformatics analysis identified RFC4 as a potential key target in OSCC progression. Additional experiments showed that RFC4 expression was significantly higher in OSCC tumor tissues than in normal tissues. Knockdown of RFC4 led to G2/M phase cell cycle arrest and inhibited the proliferation of OSCC cells both *in vitro* and *in vivo*. High RFC4 expression in OSCC tumors was linked to increased levels of MET, along with reduced levels of CD274 and CD160. Overall, the present study reveals that RFC4 may play a pivotal role in OSCC tumorigenesis and could serve as a potential predictive marker for the efficacy of immunotherapy.

## Graphical abstract

We evaluated the expression pattern and function of replication factor C subunit 4 (RFC4) and its potential as a prognostic and predictive biomarker in oral squamous cell carcinoma (OSCC) through bioinformatics analysis. Silencing RFC4 induced G2/M cell cycle arrest and suppressed OSCC cell proliferation both *in vitro* and *in vivo*. This study reveals that RFC4 may serve as a potential predictive marker of malignant proliferation of OSCC.

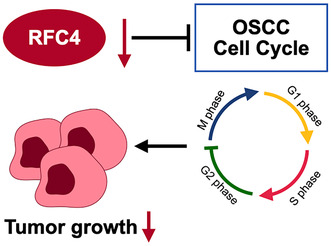

**Abbreviations**
CMSS1: cms1 ribosomal small subunit homolog
DEG: differentially expressed gene
GEO: Gene Expression Omnibus
GO: Gene Ontology
HNSCC: head and neck squamous cell carcinoma
IHC: immunohistochemistry
ITGA6: integrin subunit alpha 6
KEGG: Kyoto Encyclopedia of Genes and Genomes
NPC: nasopharyngeal carcinoma
OSCC: oral squamous cell carcinoma
OTSCC: oral tongue squamous cell carcinoma
p‐CDC2: phosphor‐cell division cycle 2
PD‐1: programmed cell death 1
PD‐L1: programmed cell death 1 ligand 1
PNO1: partner of NOB1 homolog
qRT‐PCR: quantitative reverse transcriptase‐PCR
RFC: replication factor C
TCGA: The Cancer Genome Atlas
TOM: topological overlap matrix
URB1: unhealthy ribosome protein 1
WGCNA: weighted gene co‐expression network analysis

Oral squamous cell carcinoma (OSCC) is the most prevalent type of malignant tumor in the head and neck region. It encompasses cancers that arise in the oral cavity and constitutes approximately 3–5% of all systemic malignant tumors. In recent years, significant advancements have been achieved in the detection and management of OSCC; nevertheless, the 5‐year survival rate for patients has seen minimal enhancement. OSCC is most commonly found on the tongue, followed by the gums, lips, palate, buccal mucosa and floor of the mouth. OSCC is prevalent in middle‐aged and older people (over 40 years old), especially more common in males. Risk factors for OSCC include smoking, alcohol use, betel nut chewing, infection with human papillomavirus and a family history of the disease. The incidence of OSCC has significantly increased in China over recent decades according to the Global Burden of Disease study [1], with insidious onset, high risk of metastasis, rapid progression, short survival and a high mortality rate [2].

Immunotherapy is evolving rapidly and it has emerged as an essential modality for OSCC management. Immunotherapy, with or without chemotherapy and targeted therapy, is an option for palliative systemic therapy for advanced unresectable OSCC. Surgery and radiotherapy are key curative interventions for locally advanced OSCC, with immunotherapy being an adjuvant or neoadjuvant therapy. The US Food and Drug Adminstration has approved pembrolizumab and nivolumab as treatments for recurrent or metastatic OSCC [3, 4, 5, 6, 7], with a response rate of < 50%, which is less than satisfactory. Therefore, it is imperative to explore predictive biomarkers and potential targets for the efficacy of OSCC immunotherapy.

The replication factor C (RFC) family, a complex consisting of RFC1, RFC2, RFC3, RFC4 and RFC5 subunits [8], functions as a primer recognition factor for DNA polymerase. RFCs reportedly exhibit biological activity in various malignancies and may play a crucial role in the proliferation, progression, invasion and metastasis of cancer cells. Depending on the histological and cellular characteristics of the tumor, it can function as an oncogene or a tumor suppressor gene [9]. Our previous research revealed the key role of RFC4 in nasopharyngeal carcinom (NPC) cell proliferation and NPC tumorigenesis [10]. However, the expression pattern and function of RFC4 and its potential as a prognostic and predictive biomarker in OSCC have not yet been evaluated. In this study, we focused on investigating these questions.

## Materials and methods

### OSCC data collection and differentially expressed gene (DEG) analysis

Gene expression datasets for OSCC, including GSE9844, GSE30784 and GSE31056, were retrieved from the Gene Expression Omnibus (GEO) (http://www.ncbi.nlm.nih.gov/geo) database. Additionally, gene expression and clinical data for head and neck squamous cell carcinoma (HNSCC) were sourced from The Cancer Genome Atlas (TCGA) through UCSC Xena (https://genome-cancer.ucsc.edu). The analysis of DEGs was conducted using the r package ‘limma’ [11], with a threshold established at a log_2_ fold change (FC) > 2 and *P* < 0.05.

### Weighted gene co‐expression network analysis (WGCNA) construction, biological function, and pathway annotation

To establish a co‐expression network, a total of 19 OSCC samples and nine normal controls from GSE9844, which included clinical information such as age sex, and the distribution of various immune cell types, were analyzed. The dataset for WGCNA was created by selecting genes with the highest 25% variance. The co‐expression network was built using the ‘one‐step network construction and module detection’ approach, with a soft thresholding power (β = 8) chosen based on the criteria for approximate scale‐free topology. By defining the adjacency and topological overlap matrix (TOM) and computing the associated dissimilarity (1 – TOM), we generated a gene dendrogram and identified modules, setting a minimum module size of 30. Furthermore, the correlation between module eigengenes and OSCC phenotypes was assessed.

### KEGG and GO enrichment analysis

Genes identified in the turquoise module were subjected to Gene Ontology (GO) (https://geneontology.org) and Kyoto Encyclopedia of Genes and Genomes (KEGG) (https://www.genome.jp/kegg) pathway analyses. These analyses were performed using the r package ‘clusterProfiler’ [12], applying a cutoff criterion of false discovery rate < 0.05.

### Kaplan–Meier plotter analysis

The relationship between RFC4 expression and overall survival was examined using the ‘survival’ and ‘survminer’ r packages (https://CRAN.R-project.org/package=survival), with *P* < 0.05 considered statistically significant.

### Univariate Cox analysis and multivariate Cox regression model construction

Univariable and multivariable logistic regression analyses were conducted to identify risk factors for OSCC using the ‘survival’ r package. The results were then visualized in a forest plot created with the ‘forestplot’ r package (https://CRAN.R-project.org/package=forestplot). In the multivariable logistic regression analyses, variables with *P* < 0.05 were ultimately recognized as independent risk factors for OSCC.

### Immune infiltration analysis

We calculated the proportions of 22 types of immune cells in tumor and normal tissues and groups with high and low expression of RFC4 using the CIBERSORT algorithm [13]. We visualized the correlations between the two groups using the boxplot function in r.

### Immunohistochemistry (IHC) assay

The study received approval from the Human Research Ethics Committee at Peking Union Medical College Hospital (No. I‐23PJ1849). All participants provided written informed consent, and the investigation adhered to the principles outlined in the Declaration of Helsinki. After fixation in formalin, human OSCC tissue samples and specimens from a xenograft mouse model were embedded in paraffin and sectioned into 5‐μm histological slices. The sections underwent deparaffinization and rehydration using xylene and graded alcohol, followed by heat‐induced epitope retrieval with citrate buffer to access the antigens. Subsequently, the sections were blocked with 5% BSA and incubated overnight at 4 °C with the appropriate antibodies. After washing away the primary antibodies, the sections were incubated with secondary antibodies for 90 min at 37 °C. The sections were then treated with diaminobenzidene and stained with hematoxylin. Finally, they were dehydrated, cleared, mounted, and scored. The IHC assay score was calculated using: immunoreactivity score (0–12) = RP (0–4) × SI (0–3), where RP denotes the percentage of positively stained cells and SI indicates staining intensity.

### Cell culture and transfection assay

CAL27 cells were obtained from Shanghai Malariological Technology Co., Ltd (Shanghai, China) and were cultured in RPMI 1640 medium supplemented with 10% fetal bovine serum. SCC9 cells were acquired from Shanghai Shun Run Biological Technology, Ltd (Shanghai, China), and were maintained in Dulbecco's modified Eagle's medium supplemented with 10% fetal bovine serum. Both cell lines were incubated at 37 °C in an atmosphere of 5% CO_2_. Within 10 passages post‐thawing, the cells were transfected with siRNAs or plasmids using Lipofectamine 3000 Reagent (Thermo Fisher Scientific, Waltham, MA, USA) in accordance with the manufacturer's instructions.

### RNA extraction and quantitative reverse transcriptase‐PCR (qRT‐PCR)

Total RNA was extracted from the cells using TRIzol reagent (Invitrogen, Frederick, MD, USA) to perform qRT‐PCR in accordance with the manufacturer's instructions. Briefly, the RNA was reverse‐transcribed into cDNA in accordance with the protocol of PrimeScript RT reagent kit (Vazyme, Nanjing, China). Follow the reverse‐transcription, ChamQ Universal SYBR qPCR Master Mix (Vazyme) was used to evalute target gene expression. The sequences of the target qPCR primers are listed in Table S1, and β‐actin was chosen as an endogenous control. The $2^{-\Delta \Delta C_{t}}$ technique was used to calculate the level of gene expression.

### Western blot analysis

With the addition of phosphatase and protease inhibitors, the cells were lysed in RIPA buffer (50 mm Tris–HCl at pH 7.5, 150 mm NaCl, 1 mm EDTA, 1 mm MgCl_2_ and 0.5% Triton X‐100). After measuring the total protein concentration using the BCA assay (Beyotime, Beijing, China), 20 μg of protein from each sample was loaded and separated on a 9–12% SDS/PAGE gel, then transferred onto poly(vinylidene difluoride) membranes (GE Healthcare Life Science, Chicago, IL, USA). The membranes were then blocked with 5% skim milk, and incubated with the specified primary antibodies overnight at 4 °C, followed by incubating with the appropriate secondary antibodies at room temperature for 2 h. The immunocomplexes on the membrane were visualized using an ImageQuant 800 chemiluminescent imager (Amersham plc, Amersham, UK).

The antibodies related in this experiment were listed as follows: anti‐β‐actin (dilution 1 : 10 000; sc‐47778; Santa Cruz Biotechnology, Santa Cruz, CA, USA), anti‐Phospho‐cdc2 (Tyr15) (dilution 1 : 1000, #9111; Cell Signaling Technology, Danvers, MA, USA), RFC4 (dilution 1 : 500, A5485; ABclonal, Woburn, MA, USA), cyclin B1 (dilution 1 : 1000, A2056; ABclonal), URB1 (dilution 1 : 1000, 2003‐1‐AP; Proteintech, Rosemont, IL, USA).

### Nude mouse xenograft model

Animal studies were conducted with the approval of the Animal Ethics Committee of Peking Union Medical College Hospital (MDL2022‐09‐06‐02). In brief, 6‐week‐old female BALB/c nude mice purchasing from Charles River (Beijing, China) were inoculated with 3 × 10^6^ cells via subcutaneous injection. Tumor growth was monitored every other day once it reached the appropriate size, and tumor volume was measured and calculated using: volume = π/6 length × width^2^. The mice were euthanized by cervical dislocation after 3 weeks and their tumors were excised, weighed and photographed. No mice had tumors that exceeded the maximum tumor volume threshold (2000 mm^3^). No mice had adverse outcomes and showed severe signs of illness before the experimental endpoint.

### Statistical analysis

All statistical analyses of this study were conducted via prism, version 7.0 (GraphPad Software Inc., San Diego, CA, USA) using a two‐tailed Student's *t*‐test to compare two independent groups, or one‐way analysis of variance to compare multiple independent samples. *P* < 0.05 was considered statistically significant.

## Results

### RFC4 was indicated as a hub gene in OSCC based on bioinformatics analysis

**Fig. 1:**
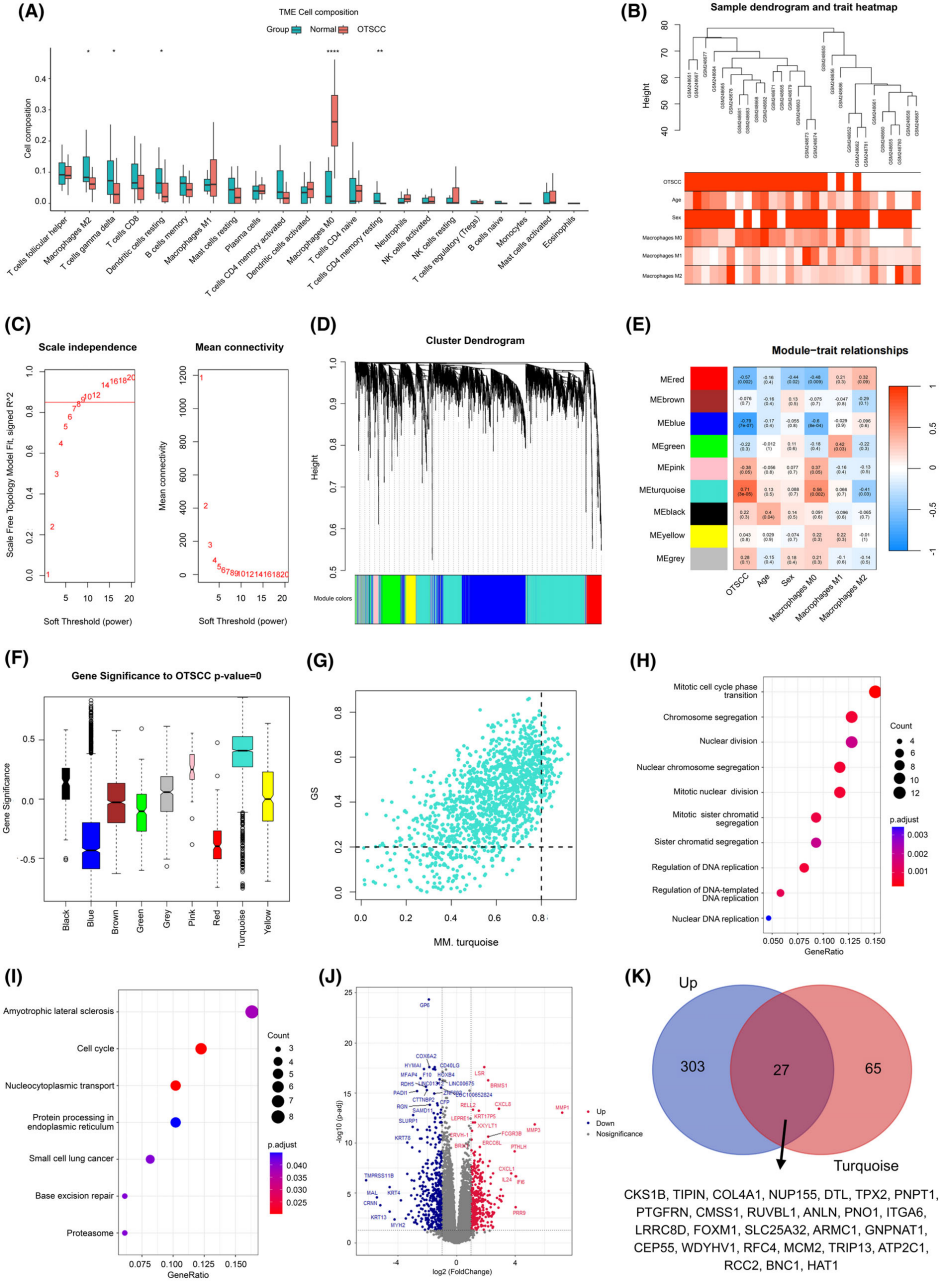
RFC4 was indicated as a hub gene in OSCC based on bioinformatics analysis. (A) The proportion of different immune cell components in OSCC tumor tissues and normal tissues through tumor microenvironment analysis. Data are expressed as thre mean ± SD (normal = 12, tumor = 26, multiple *t*‐test). (B) The sample dendrogram and trait heatmap of 19 samples of OSCC tumor and nine normal samples. (C) The appropriate soft threshold power = 8 was selected. (D) Gene cluster tree. (E) Heatmap of the correlation between modules eigengenes and clinical traits. Numbers in the upper and round brackets of each rectangle show correlation and the *P*‐value, respectively. Red represents a positive correlation, whereas blue indicates a negative correlation. (F, G) The correlation between gene significance in the turquoise module and OSCC/OTSCC. The top 10 items of GO (H) and top 7 items of KEGG (I) enrichment analysis of genes in the turquoise module. (J) A comparative analysis of gene expression between OSCC and normal tissues. (K) Venn diagram of the comparative analysis and MCODE analysis.

First, we obtained OSCC bulk transcriptional expression data from GSE9844. By comparing the proportion of different immune cell components in the tumor microenvironment of OSCC tumor tissues with those in normal tissues, we found that macrophage M2, T cell gamma delta, resting dendritic cells and resting CD4+ memory T cells were significantly decreased, whereas macrophage M0 was significantly increased (Fig. 1A) in OSCC tumor tissues. There were significant differences in macrophages between OSCC tumors and normal tissues; therefore, we used the proportion of macrophages in different subtypes to distinguish OSCC tumors from normal tissues.

Subsequently, we detected and excluded outlying samples using hierarchical clustering, leaving 19 OSCC and nine normal tissue samples for WGCNA. Thereafter, an optimal soft threshold power was calculated and chosen as eight to achieve standard scale‐free networks, whereas genes with the top 25% variance were clustered into separate modules (Fig. 1B–D). The relationships between gene modules, clinical characteristics and macrophage proportions are shown in Fig. 1E. There was a strong correlation between gene significance in the turquoise module and OSCC/oral tongue squamous cell carcinoma (OTSCC) (*r* = 0.71, *P* < 0.05) (Fig. 1F,G).

Genes in turquoise were chosen for GO_BP and KEGG enrichment analyses. The results showed that the top five enriched items in GO_BP included mitotic cell cycle phase transition, chromosome segregation, nuclear division, nuclear chromosome segregation and mitotic nuclear division, whereas the top two enriched items related to cancer included cell cycle and nucleocytoplasmic transport for KEGG (Fig. 1H,I). We performed a comparative analysis of gene expression between OSCC and normal tissues and identified 330 genes that were upregulated in OSCC. By intersecting these genes with the 92 genes in the turquoise module, we identified a subset of 27 genes. Fifteen of the 27 genes (*CKS1B*, *PNPT1*, *PTGFRN*, *CMSS1*, *RUVBL1*, *ANLN*, *PNO1*, *ITGA6*, *SLC25A32*, *ARMC1*, *GNPNAT1*, *CEP55*, *WDYHV1*, *RFC4* and *ATP2C1*) were significantly correlated with poor survival prognosis, and a total of four genes (*ITGA6*, *CMSS1*, *PNO1* and *RFC4*) had a hazard ratio > 1.5 (Fig. 1J,K), suggesting that these four genes affect survival prognosis and that their upregulation significantly increases the risk of poorer outcomes. Our previous research revealed the pivotal role of RFC4 in NPC cell proliferation and NPC tumorigenesis [10]. Based on these results, RFC4 was chosen as a potential target for further investigation.

### RFC4 was overexpressed in OSCC tumor tissues

**Fig. 2:**
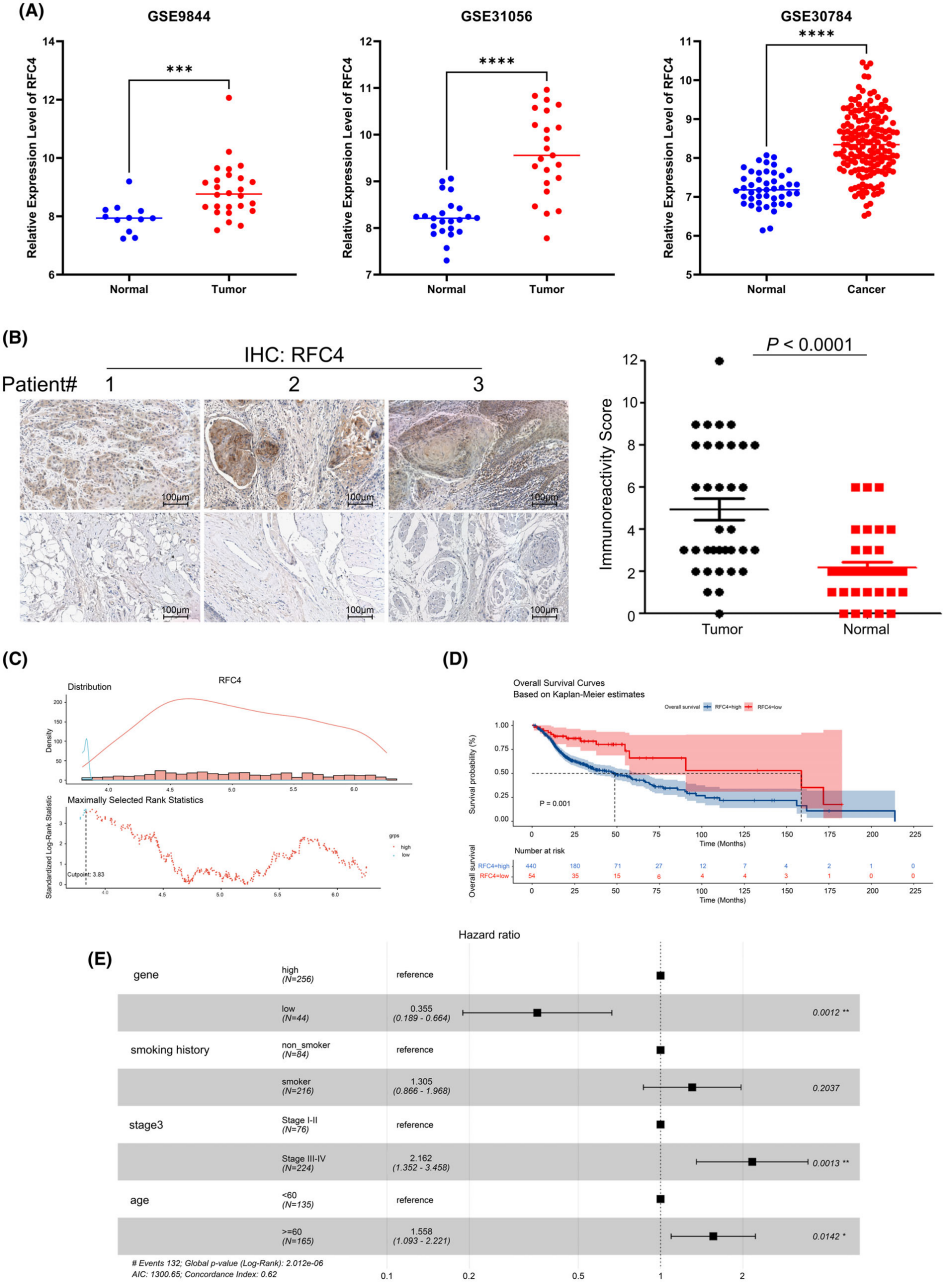
RFC4 was overexpressed in OSCC and was associated with poor prognosis. (A) The expression of RFC4 in OSCC tumor and normal tissues via analysis of data from GEO datasets. (B) Representive IHC results of RFC4 expression in normal and OSCC tumors (left). Statistic of the IHC staining of 35 OSCC samples (right). *P < 0.05, **P < 0.01, ***P < 0.001, ****P < 0.0001. Scale bars = 100 μm. (C) The survival analysis in 300 OSCC patients from the TCGA‐HNSCC dataset. (D) The expressions of RFC4 were significantly correlated with OSCC patients' survival. (E) Multivariate Cox analysis of RFC4 expression levels and clinical profiles of patients with OSCC.

Further analysis involved comparing the expression of RFC4 in normal tissues and OSCC tumor tissues by examining data from the GEO database. The results showed higher RFC4 expression in OSCC tumor tissues compared to normal tissues (Fig. 2A). To validate RFC4, a total of 35 OSCC specimens were collected for IHC analysis. The baseline clinicopathological characteristics of the patients are shown in Table 1. The IHC assay further confirmed that expression of RFC4 was significantly up‐regulates in OSCC tumor tissues compared to that in normal tissues (*P* < 0.0001), which is consistent with the results of the data analysis (Fig. 2B).

**Table 1:** Baseline demographic characteristics of 35 patients.

Characteristics	Case (*n*)
Total	35
Gender	
Male	31
Female	4
Age (years)	
< 60	2
≥ 60	9
Histology	
Squamous cell carcinoma	34
Others	1
TNM stage	
I	0
II	5
III	14
IV	15
Unknown	1

### High RFC4 expression is an independent prognostic factor for poor survival

We obtained and analyzed transcriptional expression data and correlated prognostic survival information in 300 well‐documented patients with OSCC from the TCGA‐HNSCC dataset. We determined the optimal cut‐off point for RFC4 expression (log_2_TPM) to be 3.83, which corresponded to the most significant relationship with overall survival outcome (Fig. 2C). As shown in Fig. 2D, patients with higher RFC4 expression have poorer outcomes than those with lower RFC4 expression (*P =* 0.001). RFC4 expression levels and clinical profiles of patients with OSCC were based on a univariate Cox model, which indicated that higher RFC4 expression levels (*P =* 0.0019) and TNM stage III–IV (*P* = 0.0012) were prognostic factors for poor survival, whereas age >60 years (*P* = 0.10) and positive smoking history (*P* = 0.13) were potential prognostic factors. Therefore, these four variables were included in the multivariate Cox model. The forest plot illustrated that high RFC4 expression (*P* = 0.0012), age > 60 years (*P* = 0.0142) and TNM stage III–IV (*P* = 0.0013) were independent prognostic factors for poor survival, with a global *P*‐value of 2.0126 × 10^–6^ (Fig. 2E).

### High RFC4 expression is related to the immune status of OSCC and the efficacy of immune therapy

**Fig. 3:**
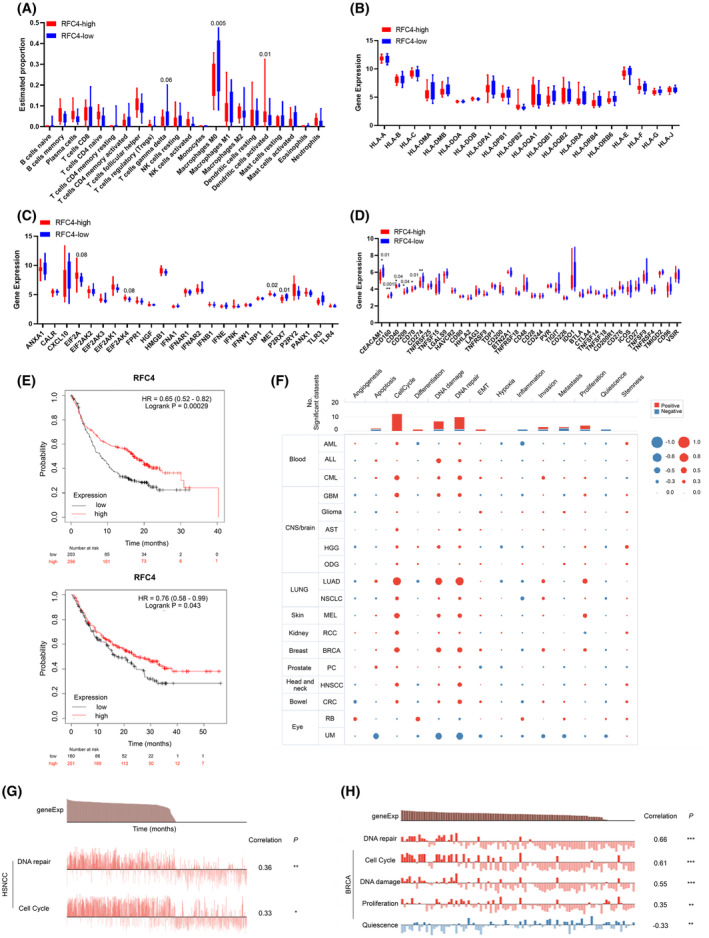
High RFC4 expression is related to the immune status of OSCC. (A) The differences in the infiltration level of 22 immune cells between low and high RFC4 groups. Data are expressed as the mean ± SD (GSE9844, tumor = 26, multiple *t*‐test). (B–D) The differences in antigen‐presenting molecules, immunogenic molecules and immune checkpoint genes between low and high RFC4 groups. Data are expressed as the mean ± SD (GSE9844, tumor = 26). (E) Kaplan–Meier plotter analysis between RFC4 expression levels with clinical outcome responds to PD‐L1 (up) and PD‐1 (down) immunotherapy. Data are expressed as the mean ± SD. (F) Single‐cell RNA‐sequencing analysis in CancerSEA. (G) Correlations between the gene of interest and functional states in different single‐cell datasets. *P < 0.05, **P < 0.01, ***P < 0.001, ****P < 0.0001.

Patients with OSCC in TCGA were stratified into two groups with high or low RFC4 expression, and the previously mentioned cutoff value was used as the demarcation threshold. The proportions of immune cells were compared between the two groups based on the bulk transcriptional data, as displayed in Fig. 3A. The group with higher RFC4 expression presented a low proportion of macrophage M0 compared to the lower RFC4 expression group (Fig. 3A). Additionally, a high level of RFC4 was found to correlate with the infiltration of active dendritic cells. Differential expression of antigen‐presenting molecules, immunogenic molecules and immune checkpoint genes was also compared between the high and low RFC4 groups. A higher level of MET and lower levels of CD274 and CD160 were found in the group with elevated RFC4 expression (Fig. 3B–D). Public data on the Kaplan–Meier plotter website (https://kmplot.com) suggest that patients with higher RFC4 expression levels respond better to programmed cell death 1 ligand 1 (PD‐L1) or programmed cell death 1 (PD‐1) immunotherapy with a preferable clinical outcome (Fig. 3E). Next, we discovered correlations between RFC4 and functional states in various cancers using single‐cell RNA‐sequencing analysis in CancerSEA (http://biocc.hrbmu.edu.cn/CancerSEA). The analysis showed the number of datasets in which RFC4 was positively related to cell cycle (12/12), DNA damage (6/7), DNA repair (9/10) and cell proliferation (3/4) (Fig. 3F). Furthermore, we found that DNA repair (*P* < 0.01) and cell cycle (*P* < 0.001), were significantly related to RFC4 in HNSCC and breast cancer (Fig. 3G).

### Knockdown of RFC4 inhibited OSCC cell proliferation *in vitro*

**Fig. 4:**
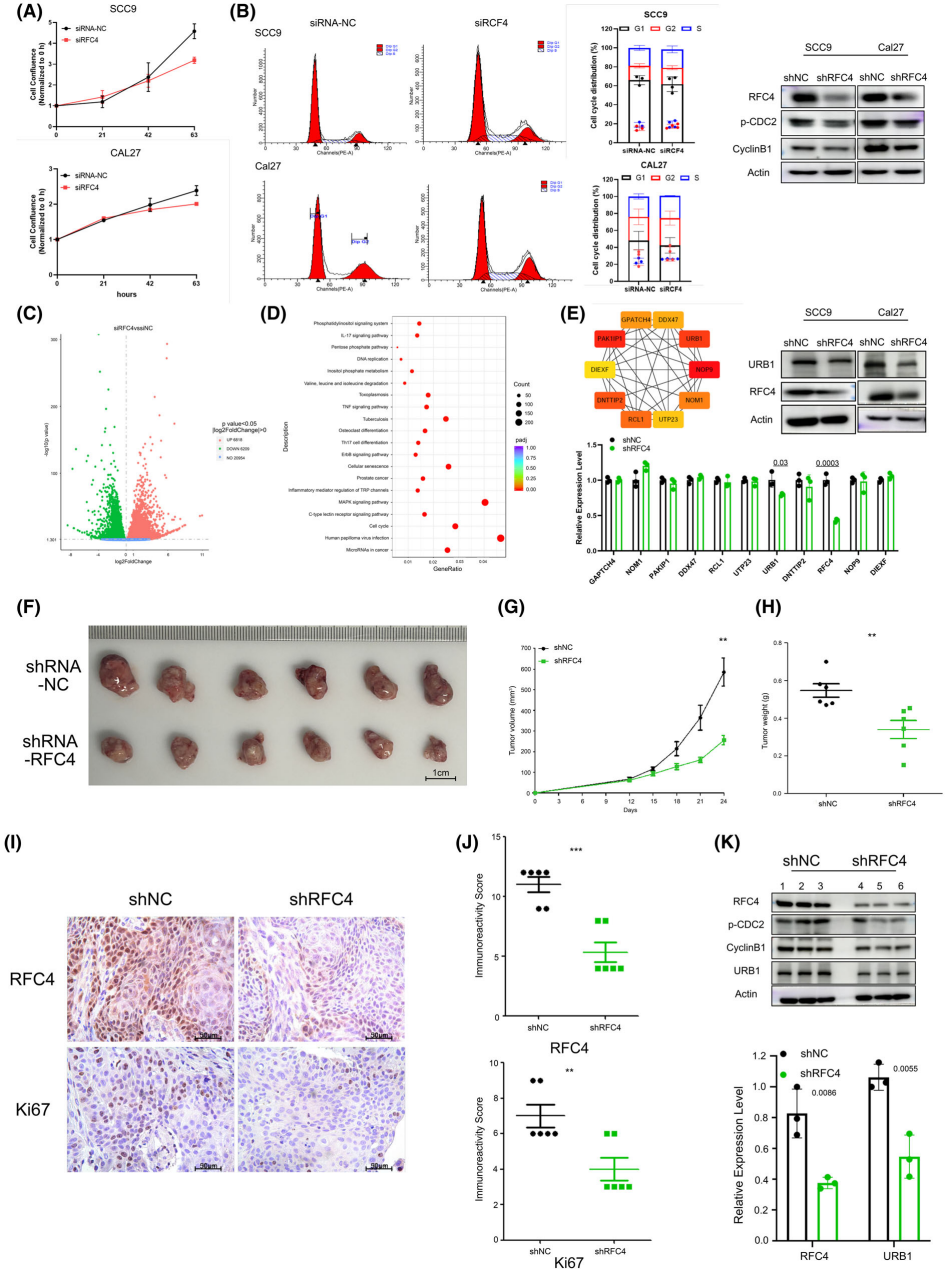
Knockdown of RFC4 inhibited OSCC cell proliferation *in vitro* and vivo. (A) The growth curve of SCC9 and CAL27 cells transfected with siRFC4 or control siNC. Data are expressed as the mean ± SEM (*n* = 3, Student's *t*‐test). (B) The cell cycle distribution after down‐regulating RFC4 in SCC9 and CAL27 cells. Statistics for the G2/M ratio in SCC9 and CAL27 cells treated with siRFC4 or control siNC. Expression of key molecules associated with the G2/M phase of the cell cycle *in vitro*. Data are expressed as the mean ± SD (*n* = 3, Student's *t*‐test). (C) Volcano plot of DEGs. (D) KEGG analysis of DEGs. (E) The top 10 hub genes via the plug‐in application of cytohubba (https://cytoscape.org/). The results of validating these genes through qRT‐PCR and western blot analysis *in vitro*. Data are expressed as the mean ± SD (*n* = 3, Student's *t*‐test). (F) Image of tumor tissues after anatomy. Scale bars = 1 cm. (G) Tumor growth curve. Data are expressed as the mean ± SEM (*n* = 6, Student's *t* test). (H) Tumor weight. Data are expressed as the mean ± SEM (*n* = 6, Student's *t*‐test). (I) Representative expression image of RFC4 and Ki67 in tumor tissues from each group. Scale bars = 50 μm. (J) Statistics for RFC4 and Ki67 expression. Data are expressed as the mean ± SEM (*n* = 6, Student's *t*‐test). (K) Expression of URB1 and other key molecules associated with the G2/M phase of the cell cycle *in vivo*. Data are expressed as the mean ± SD (*n* = 3, Student's *t* test). *P < 0.05, **P < 0.01, ***P < 0.001.

The results regarding RFC4 expression and prognosis suggest that RFC4 may be a driver of OSCC development. To further investigate the role of RFC4 in OSCC, RNA interference was applied to SCC9 and CAL27 cell lines, and cell proliferation was evaluated. The downregulation of RFC4 inhibited cell proliferation in both cell lines (*P* < 0.05) (Fig. 4A). Additionally, RFC4 siRNA transfection resulted in cell cycle arrest at the G2/M phase in the SCC9 and CAL27 cell lines (Fig. 4B). Meanwhile, p‐CDC2 and cyclin B1, key molecules associated with the G2/M phase of the cell cycle, were also expressed at low levels in the shRFC4 group *in vitro*. RNA‐sequencing was performed on both siRFC4 and siNC cell lines. DEGs were detected (Fig. 4C) and found to be enriched in specific signaling pathways (Fig. 4D), the top five of which were human papillomavirus infection, mitogen‐activated protein kinase signaling pathway, cell cycle, cellular senescence and microRNAs in cancer. These results suggest that RFC4 plays an important role in promoting cell proliferation by regulating the cell cycle in OSCC cells. A protein–protein interaction network was established using string (https://cn.string-db.org/) and visualized using cytoscape (https://cytoscape.org/). The top 10 hub genes were identified and are listed as: *NOP9*, *PAK1IP1*, *URB1*, *DNTTIP2*, *RCL1*, *NOM1*, *GPATCH4*, *DDX47*, *UTP23* and *DIEXF*, implying a potential relationship between the genes and RFC4. To further validate the findings on hub genes, qRT‐PCR and western blot experiments were performed. As shown in Fig. 4E, the mRNA and protein expression of URB1 was significantly downregulated in the shRFC4 cell lines (*P* = 0.03).

### Knockdown of RFC4 suppressed OSCC tumor growth *in vivo*

A cell line that stably expressed low levels of RFC4 (CAL27‐shRFC4) and its corresponding control cell line (CAL27‐shNC) were conducted to investigate the proliferative effect of RFC4 *in vivo*. These cell lines were transplanted into BALB/c nude mice and no significant change in body weight was observed. The results presented a significant reduction in tumor volume and weight in the shRFC4 group compared to those in the control group (*P* = 0.0015) (Fig. 4F–H). Ki‐67, a common marker of cell proliferation, was detected using IHC assays in the tumors of both groups. Histological results showed that Ki‐67 was significantly downregulated in the shRFC4 group, indicating the inhibition of tumor growth (Fig. 4I,J). The expression of URB1 mRNA in the tumor samples was then analyzed using qRT‐PCR, revealing a significant downregulation (*P* = 0.0055) (Fig. 4K), consistent with the *in vitro* results shown in Fig. 4E. Protein expressions of p‐CDC2, cyclin B1 and URB1 in tumor samples were also evaluated *in vivo*, and the findings aligned with the *in vitro* results (Fig. 4K). This suggests that downregulation of RFC4 suppresses OSCC tumor growth *in vivo*.

## Discussion

The present study found that the turquoise module was the most relevant to OSCC progression using the WGCNA method, which revealed the relationship between gene expression and the associated cellular phenotype. We further intersected the genes that were upregulated in tumor tissues and identified 27 genes aiming to determine the key genes associated with OSCC progression within the turquoise module. According to the prognostic analysis, four genes (*ITGA6*, *CMSS1*, *PNO1* and *RFC4*) affect the survival prognosis, and their upregulation significantly increases the risk of poorer outcomes. Previous studies have shown that *ITGA6* promotes the progression of laryngeal squamous cell carcinoma and enhances oxidative resistance through the Notch and Keap1/Nrf2 signaling pathways [14]; *PNO1* regulates the apoptosis of hepatocellular carcinoma cells through the mitogen‐activated protein kinase signaling pathway [15], and promotes glioma by activating THBS1/FAK/Akt signaling to promote tumorigenesis [16]. Our results validate previously reported studies, demonstrating that the WGCNA algorithm is effective in the present study. Considering our previous study on RCF4 in NPC tumorigenesis [10], we selected RFC4 as a potential target for subsequent investigation. The RFC4 protein complex is composed of five subunits with molecular weights of 140, 40, 38, 37 and 36 kDa, respectively. Previous studies have identified the role of RFC4 in cell proliferation and tumor formation, and its abnormal expression may serve as a significant prognostic indicator for various types of cancer [9, 17, 18]. However, the effects of RFC4 in OSCC progression was rarely evaluated [19]. Our study suggested that RFC4 was highly expressed in OSCC and multivariate regression analysis indicated that high RFC4 expression was an independent prognostic factor for OSCC. Our phenotypic analysis results showed that the inhibition of RFC4 expression leads to cell cycle arrest in the G2/M phase rather than in the S phase, which was corroborated by previous findings. *In vivo* tumor formation assays demonstrated that RFC4 knockdown resulted in a significant decrease in tumor growth and expression of Ki‐67 expression in mice. These results suggest that RFC4 plays a role in promoting OSCC and is a potential therapeutic target for OSCC.

In recent years, tumor immunotherapy has advanced significantly with the introduction of immune checkpoint inhibitors such as PD‐1 and CTLA‐4. Promising results for the clinical applications of these immune checkpoint inhibitors have been demonstrated, and the study of interactions between cancer‐regulating genes and immune components has become a prominent topic in cancer research [20]. Immunotherapy treatment of OSCC is less than satisfactory. The relationship between elevated RFC4 expression in tumor tissue and the presence of various types of immune cells within the tumor is essential for identifying potential predictive biomarkers for immunotherapy in OSCC. The survival of tumor cells was positively influenced by myeloid‐derived suppressor cells, which exert inhibitory effects on the proliferation of CD8 T cells and natural killer cells, both of which have antitumor capabilities [21]. Similarly, a positive correlation between RFC4 expression and the infiltration of myeloid‐derived suppressor cells, as well as a negative relation between RFC4 and natural killer T cells, was reported by Alaa Eldeen *et al*. [22], inferring that the increased expression of RFC4 may indicate an inadequate immune response toward tumor growth. In the present study, we scrutinized the increased infiltration of activated dendritic cells and decreased infiltration of macrophage M0 cells in the tumor tissues of OSCC patients with high RFC4 expression. Additionally, patients with high RFC4 expression exhibited significantly longer survival rates following immunotherapy. These results suggest that RFC4 may be a predictive marker in relation to patients' response to tumor immunotherapy. However, the regulatory mechanisms of RFC4 have not been sufficiently explored and require further investigation.

In conclusion, RFC4 was found to be overexpressed in OSCC. Silencing RFC4 inhibits OSCC cell proliferation both *in vitro* and *in vivo*. High RFC4 expression was associated with increased levels of MET, along with reduced levels of CD274 and CD160. These findings indicate that RFC4 is a potential therapeutic target in OSCC tumorigenesis and a possible predictive marker for immunotherapy efficacy.

## Conflicts of interest

The authors declare that they have no conflicts of interest.

## Author contributions

HD conceived the study and designed the experiments. PY, ZL, DW, NX, HC, XZ, SG and LW performed the experiments. ZL and SG processed, analyzed and visualized the data. HD and GW wrote the manuscript.

## Supporting information


**Table S1.** Primer sequences for real‐time qPCR.

## Data Availability

The data that support the findings of this study are available from the corresponding author upon reasonable request.
