# Supplementary material for: Knockdown of RFC4 inhibits cell proliferation of oral squamous cell carcinoma in vitro and in vivo
Source: FEBS Open Bio. 2024 Dec 13;15(2):346–58. doi: 10.1002/2211-5463.13929 (PMC11788746; doi:10.1002/2211-5463.13929)
Supplement: Supplementary file 1 — Table S1. Primer sequences for real‐time qPCR. [file FEB4-15-346-s001.docx]

**Supplementary Information Table 1.** Primer sequences for real-time qPCR

| **Gene** | **Primers (F = forward, R = reverse)** |
| --- | --- |
| **GPATCH** | F: GACTGCGGCCAACTTGGTA  R: CCACCTGAAGTCAATGTAGCC |
| **DDX47** | F: GCACCCGAGGAACACGATT  R: TCCATCCCAACTGGTCACAAG |
| **URB1** | F: TAAGTGGAGAGAAACGACCTGA  R: CGAGCCAACCTGTAACCTGA |
| **NOP9** | F: GCCCTAGCTTTGTCCACGAA  R: CGCAAGTTAGAGCGCAGAG |
| **NOM1** | F: CCACCGCAAAGACCAGACC  R: CTCCAGCTTTCGGATCTCTCG |
| **UTP23** | F: GCTTCTTCCGCAACAACTTCG  R: CCTTTCCCAATGTTTCTAGCTCT |
| **RCL** | F: ATCTGTGGAACATGACTGTAGCG  R: ATCATTGGTCACTCCTCGTAGA |
| **DNTTIP2** | F: ACTACTGCTGAATCACAGACCA  R: CAGAGGTTTCTCCATCCGTAGA |
| **DIEXF** | F: AAACTACCCACGAGCTTAAATGG  R: TGGTCTTAGTCCAGGTGGATTC |
| **PAK1IP1** | F: TATTCACCCATCTGGCAAGTTG  R: GGGGACCATTCTACTATGTGAGC |

Note: Synthesized by Tsingke Biotech, China.
